# Creating an Integrated Undergraduate Public Health Curricula: Inspiring the Next Generation to Solve Complex Public Health Issues

**DOI:** 10.3389/fpubh.2022.864891

**Published:** 2022-04-18

**Authors:** Kristin Osiecki, Jessie Barnett, Angie Mejia

**Affiliations:** Center for Learning Innovation, University of Minnesota Rochester, Rochester, MN, United States

**Keywords:** public health curriculum, undergraduate research, experiential learning, integrated courses, engaged activities

## Abstract

This article takes a novel approach of highlighting the creation and development of an integrated undergraduate public health curricula geared to students in the health sciences. In our practice, undergraduate and public health pedagogy supports innovative and proven approaches of experiential learning in our classrooms. We show how public health faculty take a team approach to teaching which has allowed them to collaborate in and outside of the classroom resulting in inherent knowledge of course materials, student engagement, and outcomes. This evolved to an overall curricula design that involves scaffolded research skills and/or projects within and between the public health courses. In addition, we highlight examples of upperclassmen utilizing these curriculum schemas outside the classroom to engage in faculty research beyond the public health discipline. This narrative describes lessons learned when teaching undergraduate students across public health curricula, how we integrated research skills within each course using pedagogical practices, and why this approach supports student engaged research within directed study and paid undergraduate research opportunities.

## Introduction: Background and Rationale for the Educational Activity Innovation

U.S. public health workplace shortages have existed for decades (1) with a chronic underfunding of public health infrastructure (2, 3). In 2020, several public health emergencies converged highlighting challenges with the United States (U.S.) health care systems, public health infrastructure, and emergency preparedness and response programs. An unprecedented pandemic, racial tensions and police violence, and extreme climate change-related disasters strained the almost $4-trillion budget allocated to health care of which only 3% is designated for public health initiatives (4). The Association of Schools and Programs of Public Health, Framing the Futures Initiative, and the accreditation by the Council on Education for Public Health (CEPH) called for the expansion of stand-alone undergraduate degree programs (5, 6), which gained momentum within the last decade. In recent years, undergraduate public health programs and degrees grew in popularity including 75,165 undergraduate public health degrees awarded from 271 institutions during 2003–2016, with over half conferred from 2011 to 2016 (5).

Undergraduate public health program frameworks, models, and standards provide recommended courses and curriculum outcomes (6–8). In 2014, literature on undergraduate public health education in the U.S. garnished only 23 articles, with two on teaching assessment and two on public health career choices (3). Recent studies support the importance of undergraduate public health degree programs emphasizing the recruitment of a diverse student body and designing curricula geared toward an undergraduate population (5, 6). From our experience, few undergraduate students can explain public health within the reflection essays assigned in the introductory public health course. With this overview, we contribute to the body of literature by mapping seven undergraduate public health courses within two sample plans: math and science or social sciences. These two plans help students choose complementary courses in a health science degree program to pursue graduate school or a career in public health professions. For example, the math and science plan meet prerequisites for environmental health, industrial hygiene, or biostatistics while the social science plan prepares students for community health promotion or maternal and child health. Public health plans are flexible so students can work with their coach to choose courses based on their needs and future goals. The public health course series incorporates experiential learning, engaged activities and high impact practices. This article examines lessons learned including unintentional missteps in curriculum design, pedagogical theory that frames our courses, and implications outside the classroom.

## Public Health and the Pedagogy of Active Learning

Numerous studies address the importance of introducing medical students to public health, health disparities, and population health curricula to address quality of care beyond individual treatment (9–12). In response to this research, the University of Minnesota Rochester (UMR) requires undergraduate health science students to take an introduction to public health course. Although some students have heard of public health, most have little or no understanding of the field. As public health course enrollment increased and sparked a demand for more classes, two public health faculty (PHF) mapped out the original four offerings scheduled annually or biennially. PHF shifted the biennial courses to annual offerings and then designed three additional courses to complete the pathway. Each course focused on core objectives with corresponding experiential learning activities. Table 1 lists the seven courses that students can enroll in as a part of the health science degree.

**Table 1:** List of undergraduate public health courses by level.

Introduction to public health (2,000-level)
Environmental health and justice (3,000-level)
Social determinants of health and health inequities (3,000-level)
Public health program immersion-(3,000-level)
Health policy and systems (3,000-level)
Introduction to epidemiology (4,000-level)
Public health research immersion-(4,000-level)

PHF regularly re-examine course objectives, discuss what worked and did not work, and adjust content based on our observations and student feedback. After 2-years, PHF took a step back and reviewed pedagogical approaches that supported student success vs. approaches that inadvertently caused a disconnect with students. For example, in the introduction to public health course, groups could choose any public health topic for the evidence-based final project. Students struggled with such broad topics, for example, obesity, and completing specific questions to create a public health intervention. Next, we utilized the American College Health Association website for topic selection with resources, but it was a stretch to extrapolate critical information to complete assessments. We combined our student-led in-class book club using Mountains Beyond Mountains by Tracy Kidder as the focus of the final project. Students interacted with the book for multiple purposes including the identification of medical and public health themes during structured in-class activities within their book club assigned groups. The consistency of engaged activities interwoven throughout the term fostered engagement within these themes allowing students to complete a succinct evidence-based project. PHF mapped curriculum meeting CEPH undergraduate competencies, removed duplicate content across the curricula, and determined integrative building blocks to reinforce public health theory and application. PHF discovered the following main themes:

- A comprehensive approach across and between the curriculum focusing on undergraduate research skills and/or studies expands student knowledge while mastering high level cognitive skills.
- Students excel with engaged public health activities at the individual level that can be scaled up to the bigger picture or institutional level topics.
- A flipped classroom design increases in-class time for active learning with the expectation of preparing for the in-class-time with online materials that are passive (but still engaged) learning.
- Students appreciate variety in course learning modalities and pacing across the undergraduate public health curriculum such as visual, auditory, reading/writing, and kinesthetic activities.
- Curriculum design can incorporate graduate level topics or concepts that tend to focus on breadth instead of depth assuming undergraduate students have mastered basic study skills.
- Students, through self-discovery, find their passion and career path through experiential learning with additional research opportunities outside the classroom in public health, social science, senior capstone experience, and directed studies.

Integrative building blocks across the public health curricula were redesigned based on an extensive literature review of undergraduate, health science, and public health pedagogy. For example, an undergraduate introduction to public health course implemented active learning experiences such as water sampling, a health behavior social media campaign, and rural access to health care plan (13). These activities were explicitly tied to course objectives (13) and provided PHF informative examples for our curricula development. A study about the European Public Health Bachelor's Program, outlined three semesters of public health courses meeting undergraduate learning objectives (14). A key program component involved a research proposal and an undergraduate senior thesis course (14). These studies helped us formulate our strategy of redesigning public health courses with undergraduate learning best practices based on Bloom's Taxonomy of Hierarchical Category Definitions and Action Verbs (BTHC), the flipped classroom, integrated undergraduate research study skills, and Kolb's Cycle of Experiential Learning (Kolb's Cycle).

Bloom's publication, the Taxonomy of Educational Objectives: The Classification of Educational Goals, is the basis of the foundational theory of cognitive skills in teaching and learning for over a half century (15). Bloom's Taxonomy created a hierarchical structure based on six levels from lowest to high levels of learning: knowledge, comprehension, application, analysis, synthesis, and evaluation (15). In 2005, this framework was revised with learning, teaching, and assessing educational objectives to include: remember, understand, apply, analyze, evaluate, and create (16). The updated Bloom's Taxonomy of Hierarchical Category Definitions and Action Verbs (BTHC) studies show that the hierarchical progression from remembering to creating supports mastery of a skill or knowledge in health care (16–19). The BTHC framework moves from the lowest level of recollection of knowledge to the highest levels of cognitive ability and is effective with public health course objectives based on these levels that correspond to our research related assessments (17). PHF discovered that a complementary pedagogical approach to BTHC is the incorporation of a flipped classroom model which increases time for procedural and metacognitive activities.

The flipped classroom is a well-studied model that supports engaged, student-centered learning that improves critical thinking and problem-solving skills (17). The flipped classroom is based upon student learning outside the classroom to prepare for in-class engaged activities focusing on problem solving, collaborative group work and working with real-world issues (20). A flipped classroom which includes lectures, videos, readings, and other passive online learning techniques are lower on the hierarchy and is then complemented with high level cognitive activities in the classroom (17). With the seven public health courses redesigned with BTHC hierarchical learning objectives, and flipped classrooms, PHF determined that each course should include research skills or student-led research studies across the curricula.

Studies show that undergraduate research is considered a high-impact educational practice and an active learning tool which is conducive to exploring complex public health issues in the classroom (21–25). Soft skills are also obtained with the ability to navigate difficult situations, increase critical thinking, and creatively solve problems (26). The research process allows students to become more resilient with the ability to overcome challenges without necessarily focusing on earning a high grade (26). Integrating undergraduate research into the curricula is mostly based on motivation at the individual faculty level (27) and is highly effective with a student-centered learning approach (28).

## A Public Health Curriculum Centered on Student Engagement

### Learning Environment

UMR is the newest campus within the University of Minnesota state-wide system that recruits and retains underrepresented students using high-impact learning practices. Our mission is to inspire transformation in higher education through innovations that empower our graduates to solve the grand health challenges. UMR offers two undergraduate degree programs-a Bachelor of Science in Health Sciences (BSHS) and a Bachelor of Science in the Health Professions (BSHP). The BSHP is offered in partnership with Mayo Clinic School of Health Sciences. Within the BSHS program, administration has created informal “career pathways” to help students navigate the wide range of careers in the field of health sciences. This is aligned with an overall institutional strategy to recruit and retain students, engage with alumni, and identify potential community partners.

UMR is a small but growing campus with approximately 1,000 students. The long-term strategic plan includes a 250% enrollment increase within the next 10 years. Currently, 40% of the student population identifies as Black, Indigenous, People of Color (BIPOC) and/or 65% as under-represented (first generation, Pell grant recipients), with 92% of students receiving some type of financial aid. Ninety-eight percentage of the 2020 graduating class completed their degree in four years or less, and the overall graduation rate is 65%. To earn a BSHS, students must complete 120 credit hours and have the unique opportunity to earn course credits at sister campuses during their senior year capstone experience.

Our public health series is grouped into the UMR public policy and global health career pathway. Due to the unique structure of our university, undergraduate minors (e.g., public health) cannot be housed within the two-degree programs. Instead, partnerships are created with the University of Minnesota Twin Cities (UMTC) because they have established programs. For example, student credits earned within the seven course public health series offered at UMR are transferable to the UMTC public health minor or can be included in the 4+1 Master of Public Health degree with UMTC School of Public Health. At this time, there is no official data about students who earned 21-credit hours in the public health pathway at graduation; however, there are discussions on gathering information to learn more about student post-undergraduate progress.

Over the past 2 years, freshmen and sophomore students are now required to take the mandatory introduction to public health course, which is a prerequisite for all the upper-level offerings. Currently, maximum enrollment with the intro course is 30 students per section for both the asynchronous online and on-ground classes. Before redesigning the public health seven-course pathway, upper-level courses had < 10 students per class. With the integrated curricula, enrollment is now 10–24 students per section with the goal of offering multiple sections in response to anticipated growth. Since the pathway is only a suggestion, students can opt to take one or more public health courses during their undergraduate career. Therefore, enrollment can be as low as five students or as high as 28 students in a class. To adjust, the flipped classroom is designed to accommodate any number of students and then engaged activities are designed to scale-up (small groups) or scale-down (individual). With large sections (60 or more students), small groups include an added peer-review component with discussions. In addition to team teaching, PHF can hire undergraduate administrative assistants (the equivalent of a graduate teaching assistant), to assist during a class.

Faculty are organized within an interdisciplinary learning center with a committee governance structure instead of a traditional department and faculty senate. Faculty in the learning center represent the physical, biological, and social sciences, math, humanities, and communication and are encouraged to guest lecture or collaborate across the disciplines. Team taught courses both within and between disciplines are offered regularly. UMRs' primary research focuses on the scholarship of teaching and learning (SoTL), while our disciplinary research investigates health inequities and environmental injustices in urban and rural communities. When proposing a new course, the modality is considered and approved by a curriculum committee that expects pedagogical justification for a blended online/in-class approach. The administration also supports increasing flexibility with student course schedules, and this blended approach requires blocking out only one class period per week instead of two. Informal and formal student assessments provide essential evidence for continual iterations of the curricula.

### Pedagogical Frameworks

PHF evaluated assessments and outcomes in relation to course objectives and redesigned them with the BTHC framework. It provided context on how students moved through the curricula taking into consideration the skill levels from lower-level courses to upper-level courses with realistic expectations of undergraduate students' abilities. With a flipped course, students completed passive online learning tasks in preparation for the in-class session. These tasks include observational and brainstorming activities, quizzes, multimedia lectures and assignments integrated into the learning management system or other education technology, such as Yellowdig, Flipgrid, or Softchalk. Online metrics evaluate student progress outside the classroom and alerts PHF to possible concepts that need to be addressed with in-class activities or with slowing down the pace of the course.

Table 2 organizes the in-class active learning BTHC action verbs that drive the scope of the activities throughout the semester.

**Table 2:** Public health course with Bloom's Taxonomy revised categorical definition and action verbs.

**Course**	**Bloom's definition**	**Course objective action verbs**
Introduction to public health	Applying	Identify, Apply, Organize, Select, Solve
Environmental health and justice	Analyzing	Theme, Relationships, Motive, Examine, Discover
Introduction to epidemiology	Analyzing	Analyze, Conclusion, Relationships, Categorize, Test for
Public health program immersion	Evaluating	Assess, Determine, Evaluate, Importance, Support, Rule on
Health policy and systems	Creating	Build, Change, Adapt, Construct, Formulate, Propose
Social determinants of health	Creating	Theory, Solve, Suppose, Discuss, Elaborate, Choose, Plan
Public health research immersion	Creating	Adapt, Build, Compile, Construct, Design, Test, Improve

A scaled-down concern is the adoption of discipline-based textbooks that are often used in both graduate and undergraduate courses. Relying on the structure of the textbook unintentionally moved students quickly through topics and concepts with an emphasis on breadth over depth. We discovered that breadth, most of the time, relied on passive learning activities (terminology, theory and concepts) to effectively cover the expansive weekly topics. From our experience during course delivery, one-on-one and group tutoring sessions, student emails, and student coursework, undergraduate students tend to search for an exact answer and struggle with drawing conclusions from a body of information. Concentrating on depth, instead of breadth, gives students an opportunity to spend more time on the specific topic with engaged materials, asking questions, and becoming more confident with problem-solving.

**Figure 1:**
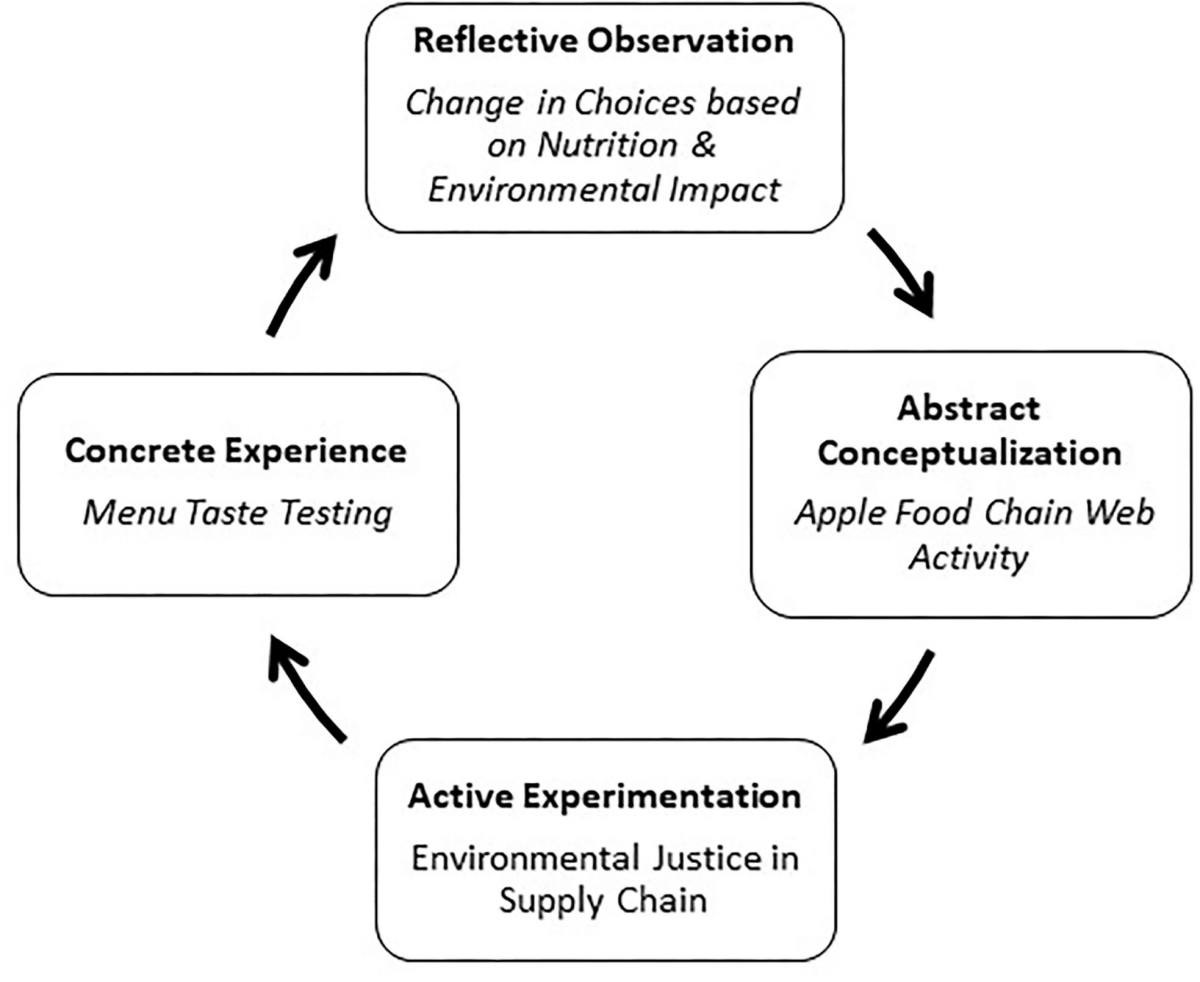
Kolb's Cycle of Experiential Learning to create in-class activities.

PHF discovered that individual level activities were instrumental in engaging and connecting students with public health concepts deeply before asking students to scale up to complex world problems and concept application. Utilizing Kolb's Cycle, each in-class session includes at least one experiential learning activity with a reflective component. The last step to our redesign is based on Kolb's Cycle. Figure 1 shows the four-stage conceptual model to design student-centric engaged activities involving concrete experience, reflective observation, abstract conceptualization, and active experimentation (29). Kolb's Cycle is based on the concept that knowledge is dependent on the transformation of experience, reflection on these experiences, and relating them back to theory to make decisions and solve problems (30). Studies show that student participation in “real-life” public health applications, including health promotion campaigns, connects students better with theory (31, 32).

For example, in the environmental health and justice course, students' blind taste a meal comparing a fast-food meat-based burger and the equivalent plant-based burger with a small glass of cow's milk, soy milk, and almond milk. It is important to note that a survey was given to students prior to this activity to tailor the menu for individual students due to possible allergies, food preferences, or religious practices, to name a few. After finishing their meal, students voted on burger #1 or #2, or beverage A, B, or C based on taste and personal preference. The results are always unexpected, and students are surprised by the outcomes. Students are then placed into five different groups and are assigned one of the menu items to investigate nutritional value and environmental impact. Each group writes their findings on the board to share with the class. The major takeaways are the two burgers only differ by 30 calories (yet have dramatically different environmental impacts), and environmental impacts of cow's milk and almond milk are much greater than soy milk (with similar nutrients). Students are asked to write a reflection on their initial choices and if they would make any changes based on the new information.

Moving onto abstract conceptualization, students engage in an apple food chain activity. Each student is given a card with a role, and they stand up to form a circle. A random student is given a ball of string and is asked to look at the other role cards to determine which one would be next in the chain. There is no right, or wrong answer and students can visually see a complex web associated with growing and eating a simple apple. With active experimentation, an apple food supply chain image is projected onto a screen (in our situation, the classroom has a projector with three screens). Students are given post-it notes and are asked to identify possible environmental justice issues that can take place along the supply chain such as farmers and laborers exposed to pesticides.

### Learning Objectives

In Table 3, each public health course has a research competency, an outcome objective, leadership role and unique type of course research skill(s) or product. Classes with a faculty research leader begin with individual level activities and then move onto group-based work. This provides a stepwise approach to critical thinking and problem solving before introducing group social dynamics. Student project leaders work within research groups on collaborative research activities in a supervised classroom. This allows PHF to engage with groups to answer questions, navigate research obstacles, and keep the groups on task. The positive experience of implementing an empowering classroom research study, especially a study in which students investigated a problem that directly impacted them, cannot be understated.

**Table 3:** Public health course research strategy.

**Course**	**Type of assessment**	**Research leader**	**Content**	**Outcome**
Introduction to public health	Individual	Instructor	Evidence-based public health	Identify problem, etiology, recommendation, interventions and evaluation (PERIE Process). Utilize CDC best practices
Environmental health and justice	Group	Instructor	Health behavior model, experiments, annotating book chapters	Practice annotation research skills, collaboration, and community-based learning
Introduction to epidemiology	Individual	Instructor	Evidence based public health, case studies, analyzing disease surveillance data	Strengthen quantitative skills in descriptive statistics, public health policy, and disease surveillance
Public health program immersion	Group/ Individual	Student	Public health program evaluation research	Create and implement a public health program with high school mentees within an outdoor field experience
Health policy and systems	Group	Student	Qualitative research on university level policy	Design qualitative research study with photovoice, interviews, coding, themes, and findings
Social determinants of health	Individual	Instructor	Literature review and applying theory of change and logic model to public health research	Interpret peer reviewed literature, design a project proposal, incorporate Theory of Change and Logic Model into program processes
Public health research immersion	Group/ Individual	Student	Mixed methods research	Design and implement a research study based on an outdoor field experience

## Collecting, Analyzing, and Evaluating Courses and Assessments

Data collected in public health courses, including student assessments, reflections, surveys or any deliverable produced during the semester are included in an institutional IRB for SoTL research. PHF creates materials for qualitative research initiatives looking at student study skills such as close reading and annotating, student engagement with their peers, and student critical thinking skills with complex issues. Below are some examples of our current data collection.

Although not always an optimal resource for evaluation, we track student rating of teaching scores (SRTs) and corresponding comments. Overall, public health course SRTs have increased to include only positive affirmations of agree and strongly agree in each category. In addition, the feedback provides student insights of their learning experiences and course criticisms such as:

- “Taught me a lot about qualitative research and showed me how to be creative in my research”.
- “She challenged us to look at concepts from different viewpoints”.
- “I would like to have done more interviews with students for our research project”.
- “I would like more time working with my group in class”.

PHF integrates constructive criticism such as simplifying research steps (expecting them to complete too much on their own), modifying the data collection to balance student needs with feasibility, or restructuring in-class time to support more group work.

Across the seven-course public health curriculum, at least one assessment includes close reading skills and annotating text. For example, in the immersion classes, students borrow their textbooks from the class resource pool and are given four different colored sticky notes. Each sticky note represents a theme (i.e., public health or individual connection) for students to annotate within the book. Students return their textbooks with the sticky notes. PHF code and analyze student engagement with the materials, depth of knowledge, and thematic interests using students' annotations. In another instance, the students read chapters embedded into the Hypothes.is app. Students can highlight, annotate, and comment on each other's annotations to collaborate on a group worksheet in Google Docs. With the suggested editing tool on, faculty can see the individual contributions to the worksheet. Both documents are analyzed using a qualitative time/space software which tracks how students interact with each other.

We are collecting longitudinal data examining student engagement with Yellowdig, a community building platform, that includes a variety of learning tools students use to earn points toward a weekly goal. This design encourages student interactions with little influence from instructors who monitor the metrics behind the scenes. The conversations are deidentified and then analyzed for themes within and between cohorts to determine their engagement with both individual level and big picture public health problems.

Other types of data collection include focus groups, pre and post surveys, semester journals, or handwritten weekly reflections. Assessments associated with active learning such as group white board work or venturing out into the community are photographed to capture the experiences. The objective is to holistically view all activities to determine if there are different components that might evolve into a SoTL study–and to evaluate student engagement across the curricula.

## An Unexpected Undergraduate Research Evolution

The integrated public health curricula has piqued student research interests outside the classroom. Students ask to participate in public health research through institutional undergraduate research stipends, applying for undergraduate research assistantships or earning directed study credits. Students can also request a research mentor in other disciplines to pursue their own research initiatives. To support undergraduate researchers' efforts, it takes more time to prepare, engage, and complete research tasks as undergraduate students gain confidence with their research skills while applying them in “a real-life setting.”

The research trajectory of three students, D, C, & H that completed public health course(s) exemplify potential long-term implications for supporting undergraduate student opportunities outside the classroom. D wanted to learn more about reproductive health and completed a directed study with Dr. Osiecki and Dr. Barnett. D was interested in a transnational phenomenon while also exploring more specialized qualitative approaches (such as grounded theory, ethnography, or narrative analysis.) and was introduced to Dr. Mejia, sociology faculty. D started working on Dr. Mejia's ongoing research project examining reproductive health attitudes of undergraduate students. D organized and collected texts and interviews into a qualitative data set while learning Atlas.Ti software. By the third week of the term, D was ready to work with Dr. Mejia on the basics of grounded theory analysis. Dr. Mejia then hired D as an undergraduate research assistant to facilitate focus groups and collect in-depth interviews for her project on COVID-19 perspectives of Women of Color on campus.

C expressed interest in learning more about community-based participatory research (CBPR) methods. C spent 1 year working with Dr. Osiecki on an environmental justice study. C then collaborated with Dr. Mejia and a community partner, the Village Community Garden and Learning Center, to collect data (focus groups with BIPOC refugee farmers of SE Asian descent) and train other volunteers on research tasks. Dr. Mejia then hired C as an undergraduate research coordinator for a project on women and STEM in mental health. (Now an alumna, C is a member of the board of directors of the above-referenced community partner).

H spent 2 years with Dr. Osiecki and Dr. Barnett outside the classroom as an undergraduate assistant supporting the public health immersion courses including finding and writing grants for the field experiences. In her senior year, H signed up for directed study hours with Dr. Mejia to develop a funding strategy to help a small agricultural cooperative. H, was able to apply her previous public health skills to write several small grant proposals while actively participating with the agricultural cooperative. Out of three proposals written by the student, one was given comments by the grantor with an invitation to re-apply, and another one was fully funded with an invitation to submit to another grant call for applications. The fully funded proposal allowed for the small agricultural cooperative to receive funds and in-kind donations of building materials. The cooperative was able to set up a system of pest control barriers for the growers while also providing funds to develop a week-long summer program for K-12 students to learn about human consumption patterns.

D and Dr. Mejia wrote a chapter on reproductive health in the classroom and submitted it to a call for papers for an edited volume on the health humanities. (As of writing this article, the chapter has been accepted with minor revisions). C co-authored a collaborative autoethnography (as a second author) with Dr. Mejia as a corresponding author on COVID-19 and health science undergraduates' emotional health. Student D and C, along with the third author, submitted another paper looking at care work and caregiving inequities along intersectional lines and COVID-19. As of this date, the paper has received a revise and resubmit with minor changes.

Students D, C, and H, treated as research collaborators, instead of task masters, show high levels of work while gaining confidence in their abilities. A crucial component to this unexpected success is the informal conversations between PHF and their sociologist colleague. Sharing of ideas, teaching methods, and best practices started as invitations to guest lecture in each other's classes and evolved into a larger undergraduate student research initiative. Currently, Dr. Osiecki and Dr. Mejia are working together on campus food insecurity with undergraduate research assistants. Dr. Osiecki, Dr. Barnett and Dr. Mejia are also writing a student mentorship article, and we are brainstorming possible research opportunities with biology and fine arts faculty. It is the shared undergraduate student research experiences that brought us all together.

## Realities Incorporating Undergraduate Research Into Public Health Curricula

The biggest challenge of implementing an integrated research curriculum is time. Incorporating research skills and student-led research projects requires PHF planning to manage student time in and outside the classroom. Research skill courses require a slower pace with easy-to-follow instructions in the flipped classroom environment. Research study courses need flexibility and adaptability when students change directions, as well as realistic expectations for research with regards to limitations presented by a semester's finite length. There are additional demands of meeting with students outside the classroom during office hours or by appointment. The outcomes support the additional time, and with continual course offerings and iterations, PHF becomes more efficient the instruction.

Planning to facilitate student research is different from planning research for students. PHF relies on looser constructs to readjust in-class activities due to the increased uncertainty involved with undergraduate student research needs. To prepare for these readjustments, activities are planned for the overall assessment and not for a particular class session. Predicting length of engagement within a particular task is difficult with some activities going faster or slower than expected. We also over plan with the ability to change directions based on student engagement levels. Depending on the time of day or week within a semester, students can exhibit high or low energy and pushing students is not necessarily the answer. Also, when students show commitment and connections with an activity that is above and beyond expectations, PHF may adjust the schedule to enable sustained engagement.

Lastly, team teaching is hard to do. It is not a division of responsibilities or rotating materials but instead learning how to complement each other in the classroom. Navigating group work and discussions requires a rhythm, PHF banter off each other and share complementary expertise, and it is a key to understanding body language and moving around the class. Both parties need to be dedicated to the model and continue working together on curriculum outside the classroom to be on the same page.

## It's All About What Works for You

This integrated public health curricula works for us because of unique institutional qualities that support PHF team teaching, smaller classroom sizes, and SoTL faculty research agendas. We acknowledge that academia looks different depending on the size of the institution, college, and department with different expectations of teaching, research, and service. Active learning, undergraduate research, and integrating public health curricula is not an all or nothing endeavor. Within an individual course, choose an objective with a higher-level outcome to pair with an in-class engaged activity. In large lecture halls, students can pair up, turn to the row behind them, or even move into the hallway. In a classroom, encourage students to move around desks or work on chalk/white boards.

If collaborating or team teaching is appealing, find a colleague within or outside the department, who would be interested in guest lecturing with you. The planning time commitment is low with potential high rewards. For example, PHF guest lectures with colleagues in other disciplines to create activities on topics such as food insecurity or realities of being a low-wage earner. It is an opportunity to watch and learn from each other within different contexts. Collaborating between two or more classes, can also be a synergistic fit, for example, introducing intersectionality in a sociology course and then examining population characteristic and social determinants of health within the praxis of intersectionality. The goal is to support learning that benefits both faculty and students.

Lastly, undergraduate research within courses is a high impact practice with a lot of flexibility to meet course objectives. The type of research or skill, how students engage with each other, the duration of the project, and instructor facilitated activities, to name a few, can be designed to be engaged activities, assessments, or both. It is also an opportunity to bring inspiring faculty research into the classroom to expose students to discipline-based public health studies that take place outside the classroom. Public health curricula lend itself to higher level learning approaches and PHF embraced theories and models that worked best for them.

## Data Availability Statement

The original contributions presented in the study are included in the article/supplementary material, further inquiries can be directed to the corresponding author/s.

## Author Contributions

KO and JB contributed to conception and design of the curriculum, pedagogical research, and data collection. AM and KO contributed to undergraduate research theory and application. KO, JB, and AM collaborated and wrote sections of the manuscript. All authors contributed to manuscript revision, read, and approved the submitted version.

## Conflict of Interest

The authors declare that the research was conducted in the absence of any commercial or financial relationships that could be construed as a potential conflict of interest.

## Publisher's Note

All claims expressed in this article are solely those of the authors and do not necessarily represent those of their affiliated organizations, or those of the publisher, the editors and the reviewers. Any product that may be evaluated in this article, or claim that may be made by its manufacturer, is not guaranteed or endorsed by the publisher.

## References

[1] DeSalvo K Parekh A Hoagland GW Dilley A Kaiman S Hines M . Developing a financing system to support public health infrastructure. Am J Public Health. (2019)10:1358–61. 10.2105/AJPH.2019.305214 31415208 PMC6727291

[2] Bekemeier B Marlowe J Squires LS Tebaldi J Park S. Perceived need versus current spending: gaps in providing foundational public health services in communities. J Public Health Manag Pract. (2018) 24:271–80. 10.1097/PHH.0000000000000612 28832431

[3] Evashwick CJ Tao D Arnold LD. The peer-reviewed literature on undergraduate education for public health in the United States, 2004-2014. Front Public Health. (2014) 2:223. 10.3389/fpubh.2014.00223 25453028 PMC4233906

[4] Tulenko K Vervoort D. Cracks in the system: the effects of the coronavirus pandemic on public health systems. Am Rev Public Adm. (2020) 6–7:455–66. 10.1177/0275074020941667

[5] Recommended critical component elements of an… [Internet]. Association of Schools and Programs of Public Health (2012). Available online at: https://aspph-wp-production.s3.us-east-1.amazonaws.com/app/uploads/2014/04/CCE_2012-08-03-FINAL.pdf (accessed January 26, 2022).

[6] Resnick B Leider J Riegelman R. The landscape of US undergraduate public health education. Public Health Rep. (2018) 133:619–28. 10.1177/0033354918784911 30084738 PMC6134569

[7] Wykoff R Petersen D Weist EM. The recommended critical component elements of an undergraduate major in public health. Public Health Rep. (2013) 128:421–4. 10.1177/003335491312800516 23997294 PMC3743296

[8] Mowry D Abels P. An approach to development of a public health minor. Peer Rev. (2009) 11:23.

[9] Institute of Medicine. Unequal Treatment: Confronting Racial and Ethnic Disparities in Health Care. Washington, DC: National Academies Press (2002). p. 31. 25032386

[10] Godfrey S Nickerson K Amiel J Lebwohl B. Development of an online public health curriculum for medical students: the public health commute. BMC Med Educ. (2019) 19:298. 10.1186/s12909-019-1734-4 31376832 PMC6679425

[11] Berwick DM Finkelstein JA. Preparing medical students for the continual improvement of health and health care: Abraham Flexner and the new “public interest”. Acad Med. (2010) 85:S56–65. 10.1097/ACM.0b013e3181ead779 20736631

[12] Haq C Stearns M Brill J Crouse B Foertsch J Knox K . Training in urban medicine and public health. Acad Med. (2013) 88:352–63. 10.1097/ACM.0b013e3182811a75 23348092

[13] Yeatts K. Active learning by design: an undergraduate introductory public health course. Front Public Health. (2014) 2:284. 10.3389/fpubh.2014.00284 25566526 PMC4275053

[14] Kuiper T Meijer A Moust J. Innovation in public health teaching: the maastricht experience. Public Health Rev. (2011) 33:300–14. 10.1007/BF03391635

[15] Bloom BS. Taxonomy of educational objectives: the classification of educational goals. New York, NY: Longmans, Green (1956).

[16] Anderson LW Krathwohl DR. A Taxonomy For Learning, Teaching, and Assessing: a Revision of Bloom's Taxonomy of Educational Objectives. New York, NY: Longmans (2001).

[17] Morton DA Colbert-Getz JM. Measuring the impact of the flipped anatomy classroom: The importance of categorizing an assessment by Bloom's taxonomy. Anat Sci Educ. (2017) 10:170–5. 10.1002/ase.1635 27427860

[18] Mashamba-Thompson TP Sartorius B Stevens FC Drain PK. Experiential Bloom's Taxonomy learning framework for point-of-care diagnostics training of primary healthcare workers. Afr J Lab Med. (2016) 5:1–4. 10.4102/ajlm.v5i1.449 28879117 PMC5436408

[19] Sharunova A Butt M Qureshi AJ. Transdisciplinary design education for engineering undergraduates: mapping of bloom's taxonomy cognitive domain across design stages. Procedia CIRP. (2018) 70:313–8. 10.1016/j.procir.2018.02.042

[20] Hawks SJ. The flipped classroom: now or never? AANA J. (2014) 82:264–9. 25167605

[21] Beckman M Hensel N. Making explicit the implicit: Defining undergraduate research. CUR Quarterly. (2009) 4:40–4.

[22] Laursen S Hunter AB Seymour E Thiry H Melton G. Undergraduate research in the sciences: Engaging students in real science. New York: Jossey-Bass (2010).

[23] Levy P Petrulis R. How do first-year university students experience inquiry and research, and what are the implications for the practice of inquiry-based learning? Stud High Educ. (2012) 37:85–101. 10.1080/03075079.2010.499166

[24] Lopatto D. Science in Solution: The Impact of Undergraduate Research on Student Learning. Tucson: Research Corporation for Science Advancement (2009).

[25] Steinberg SR Kincheloe JL. Students as Researchers: Creating Classrooms that Matter. London: Routledge Falmer (1998).

[26] Wolf SL. Undergraduate Research as Engaged Student Learning. New Directions for Teaching and Learning. (2018) n154:75–85. 10.1002/tl.20293

[27] Griffiths R. Knowledge production and the research-teaching nexus: The case of the built environment disciplines. Stud High Educ. (2004) 29:709–26. 10.1080/0307507042000287212

[28] Kember D. A reconceptualisation of the research into university academics' conceptions of teaching. Learning Instr. (1997) 7:255–75. 10.1016/S0959-4752(96)00028-X

[29] Hartley MP. Experiential learning using Kolb's cycle of learning. J Nurs Educ. (2010) 49:120. 10.3928/01484834-20100119-02 20143767

[30] Arbuthnot E Hansen-Ketchum P Jewers H Moseley J Wilson C. Bringing theory to life: Engaging nursing students in a collaborative population-based screening project. Int J Nurs Educ Scholarsh. (2007) 4:Article3. 10.2202/1548-923X.1358 17402929

[31] Gabbay John. Courses of action—the case for experiential learning programmes in public health. Public Health. (1991) 105:39–50. 10.1016/S0033-3506(05)80315-4 2008502

[32] Roman V. Experiential learning in undergraduate education–doing and reflecting. Am J Med Sci. (2018) 356:88. 10.1016/j.amjms.2018.06.001 30219164

